# High Serum Levels of Malondialdehyde and 8-OHdG are both Associated with Early Cognitive Impairment in Patients with Acute Ischaemic Stroke

**DOI:** 10.1038/s41598-017-09988-3

**Published:** 2017-08-25

**Authors:** Zhihua Liu, Yuntao Liu, Xinjie Tu, Huiping Shen, Huihua Qiu, Huijun Chen, Jincai He

**Affiliations:** Department of Neurology, the First Affiliated Hospital of Wenzhou Medical University, Wenzhou, 325000 China

## Abstract

Post-stroke cognitive impairment (PSCI) is an increasingly prevalent sequel after stroke that may associate with poor functional outcome and increased risk of recurrent stroke. We aimed to explore the relationship between oxidative stress biomarkers and the presence of PSCI. 193 first-ever acute ischaemic stroke patients were consecutively enrolled in the current study. The oxidative stress biomarkers malondialdehyde (MDA) and 8-hydroxydeoxyquanosine (8-OHdG) were measured within 24 h after admission. Cognition function was evaluated by the Mini-Mental State Examination (MMSE) at 1 month after stroke. Serum levels of 8-OHdG and MDA were both significantly higher in the PSCI (*p* < 0.001) compared with the non-PSCI group. Both the serum levels of both 8-OHdG and MDA were negatively correlated with the MMSE score. Receiver operating characteristic curve analysis was used to evaluate 8-OHdG and MDA as markers of a high risk of PSCI and produced area under curve values of 0.700 and 0.793. Adjusted logistic regression showed that serum 8-OHdG and MDA levels remained as independent markers of PSCI. High serum levels of malondialdehyde and 8-OHdG are associated with the presence of PSCI at 1 month after stroke.

## Introduction

Cognitive impairment is an increasingly prevalent sequel after stroke^1^, even in those with successful clinical recovery^2^. The diagnosis of post-stroke cognitive impairment (PSCI) deserves wide attention because the presence of cognitive impairment has been associated with poor functional outcome^2^ and a higher risk of future stroke^3^. However, traditional known risk factors of PSCI, including older age, sex differences and a previous history of stroke^4^, are not readily amenable to treatment. Therefore, it is essential to identify pathophysiological mechanisms that may result in PSCI, especially in the early stage of stroke; however, research into the biochemical changes in PSCI is still not systematic and is lacking in both quality and quantity.

Increasing evidence has demonstrated the vital role of oxidative stress pathways in the regulation of cognitive impairment related processes, including vascular inflammation^5^, neurodegeneration^6^, and blood–brain barrier (BBB) dysfunction^7^, all of which indicate the potential relationship between oxidative stress pathways and cognitive impairment.

The oxidative stress pathway can produce oxidative damage of lipids and nucleic acids. 8-hydroxydeoxyquanosine (8-OHdG) and malondialdehyde (MDA) are common end-products of deoxyribonucleic acid (DNA) oxidation and lipid peroxidation, respectively, and can be detected in body fluid or brain in humans^8^. The systemic oxidative stress response to acute ischaemic stroke involves increases in 8-OHdG and MDA, which have also been associated with poor functional recovery^9^ and mortality^10^. While higher peripheral blood levels of 8-OHdG^11^ and MDA^12^ have been associated with dementia, and some researchers have found that oxidative stress is related to cognition, without dementia or stroke^13^, the association between 8-OHdG or MDA and PSCI has not been investigated.

Therefore, we measured serum levels of 8-OHdG and MDA within 24 h after admission in patients with acute ischaemic stroke, to determine whether oxidation products are associated with the presence of PSCI, and if so, at what concentrations.

## Results

### Baseline characteristics of the study participants

**Figure 1:**
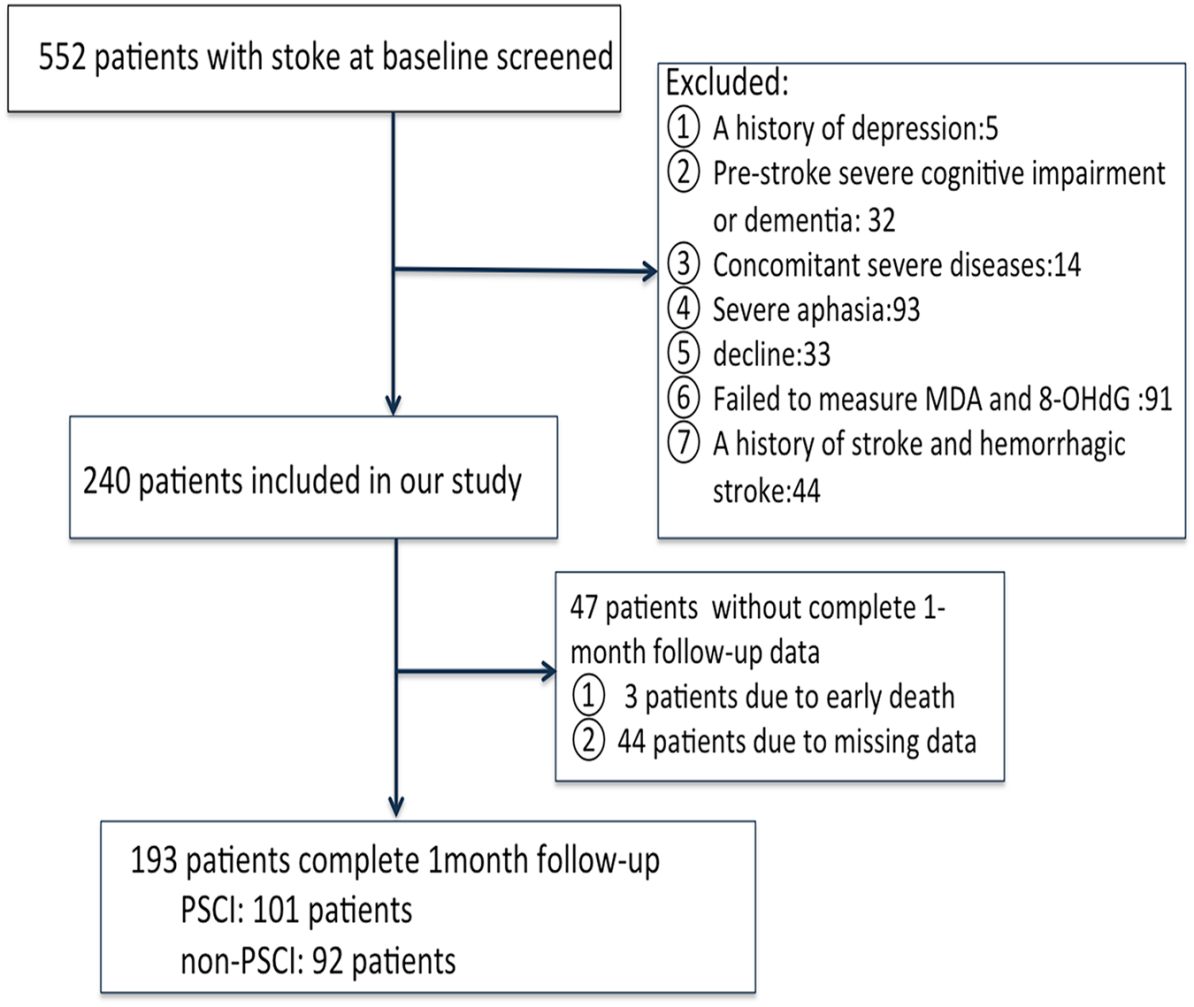
Study recruitment profile. PSCI, post-stroke cognitive impairment.

During the inclusion period, a total of 552 consecutively admitted acute stroke patients were screened, and 240 patients who met entry criteria were registered (Fig. 1). By the 1-month follow-up, 47 (19.6%) of the patients had dropped out of the study; 193 patients were included in our study. There were no significant differences between the groups in terms of baseline characteristics such as age, sex, hypertension, diabetes mellitus, NIHSS score and serum levels of MDA and 8-OHdG (see Supplementary Table S1). Drug treatments for the recruited patients in our study are presented in Table 1 and Supplementary Table S6.

**Table 1:** Baseline characteristics of the studied sample.

Baseline characteristics	PSCI Patients (n = 101)	Non-PSCI Patients (n = 92)	*P* value
**Demographic characteristics**			
Gender, (M/F)	56/45^a^	65/27	0.029
Age, yr, median (IQR)	66 (56–72)	60 (52.3–65.8)	0.001
Symptoms onset to hospital (IQR, days)	3 (1–5)	3 (1–7)	0.399
Years of education (years)	4 (0–7.5)	4 (1–7)	0.756
Education level,n (%)			0.345
Illiterate	26 (25.7)	22 (23.9)	
Primary school	36 (35.6)	45 (48.9)	
Secondary school or above	39 (38.6)	25 (27.1)	
SBP (mmHg)	156.8 ± 23.20	152.7 ± 20.97	0.213
DBP (mmHg),	82.0 (74.0–91.8)	82.0 (75.0–90.0)	0.828
BMI, kg/m^2^	24.2 (22.1–26.0)	24.1 (21.7–26.3)	0.688
**Drugs used chronically prior to the ischemic events**			
Hypertension medicine use, n (%)	60 (59.4)	37 (40.2)	0.008
Diabetes medicine use, n (%)	17 (16.8)	16 (17.4)	0.918
Lipid-lowering medicine use, n (%)	0	1 (1.1)	0.967
Aspirin or clopidogrel use, n,(%)	7 (6.9)	5 (5.4)	0.667
Stroke etiology,n (%)			0.063
Cardioembolism	8 (7.9)	2 (2.2)	
Atherosclerosis	91 (90.1)	83 (90.2)	
Small vessel occlusion	2 (2.0)	6 (6.5)	
Other undetermined etiology	0	1 (1.1)	
Hypertension	72 (71.3)	52 (56.5)	0.033
Diabetes mellitus	37 (36.6)	27 (29.3)	0.543
Coronary artery disease	1 (1.0)	5 (5.4)	0.173
Hyperlipidemia	67 (66.3)	62 (67.3)	0.328
Smokers	45 (44.6)	36 (39.1)	0.446
Alcohol consumers	38 (37.6)	38 (41.3)	0.601
NIHSS score at admission	3 (1–6)	2 (1–4)	0.019
BI score at discharge	85 (55–100)	95 (72–100)	0.010
PSQI score at discharge	5 (3–8)	4 (2–6)	0.029
HAMD-17 score at discharge	6 (3–9)	3 (1–6)	0.002

Continuous variables are expressed as the mean ± standard deviation (SD) or the median (interquartile range). Categorical values are given as frequencies (percentages).

The *p* values reflect comparisons between PSCI group and non-PSCI group.

Abbreviation: BMI, body mass index; SBP, Systolic blood pressure; DBP, Diastolic blood pressure; NIHSS, National Institutes of Health Stroke Scale; BI, modified Barthel Index; PSQI, Pittsburgh Sleep Quality Index;HAMD-17, Hamilton depression rating scale 17-item; PSCI, Post-stroke cognitive impairment.

In the study population, 101 patients (52.3%) were diagnosed with PSCI 1 month after admission. Patients with PSCI (illiterate excluded) were divided into mild cognitive impairment (n = 21, 28%) and moderate to severe cognitive impairment (n = 54, 72%) groups. The basic characteristics of the study subjects are presented in Table 1. Compared to non-PSCI, PSCI patients were significantly older (*p* = 0.001) and had higher NIHSS scores (*p* = 0.019), HAMD scores (*p* = 0.002), PSQI scores (*p* = 0.029) and lower BI scores (*p* = 0.010). Meanwhile, patients with PSCI were more likely to be male (*p* = 0.029) and have hypertension (*p* = 0.033). No significant differences were found in years of education, stroke aetiology, and other vascular risk factors, such as smoking, drinking, diabetes mellitus, coronary artery disease and hyperlipidaemia, between the PSCI and non-PSCI groups.

### Higher serum levels of 8-OHdG and MDA are associated with cognitive impairment 1 month after stroke

Serum MDA and 8-OHdG levels were stratified according to the number of days elapsed from the time of symptoms onset to hospital admission (see Supplementary Fig. S1 and Supplementary Table S2). However, we found that there were no significant differences between groups.

Infarct volume and lesion location of patients for whom diffusion-weighted imaging (DWI) data were available (n = 120) are shown in Supplementary Table S3. The infarct volume in total stroke patients was 1411.34 (599.94–4194.35) mm^3^. The infarct volume in patients with PSCI was larger than patients without PSCI [1599.82 (701.78–7725.20) vs. 1175.49 (413.67–2710.17) mm^3^, *p* = 0.039] (see Supplementary Table S3). Patients who had undergone DWI were divided into four groups according to infarct volume (see Supplementary Table S4). However, there were no significant differences in MDA (*p* = 0.697) and 8-OHdG levels, (*p* = 0.912) according to infarct volume.

In addition, we compared differences in MDA and 8-OHdG levels according to the location of the ischaemic event in those 120 patients for whom DWI data were available (see Supplementary Table S5). Serum MDA levels were higher in patients with left hemispheric infarction than in patients with right hemispheric infarction (*p* = 0.023). However, there were no significant differences in serum 8-OHdG levels according to different infarction locations.

**Figure 2:**
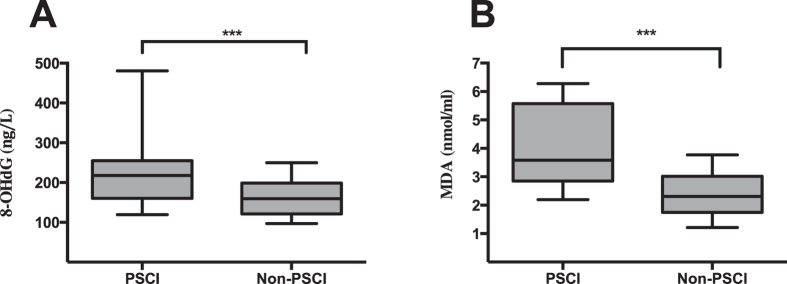
Comparisons of serum 8-OHdG and MDA levels in patients with PSCI and patients without cognitive impairment. (**A**) 8-OHdG levels, (**B**) MDA levels. In the box-and-whisker plots, the horizontal line in the middle of each box indicates the median value; the lower and upper ends of the box represent the 25th and 75th percentiles, and the peripheral lines extending to the outer fences represent 10th and 90th percentiles, respectively. ***p < 0.001 compared with the non-PSCI group via Mann- Whitney U tes Abbreviation: 8-OHdG, 8-hydroxydeoxyquanosine; MDA, malondialdehyde; PSCI, post-stroke cognitive impairment.

Serum 8-OHdG levels were significantly higher in the PSCI than in the non-PSCI group (217.5 (160.3–254.8) vs. 159.4 (120.8–198.6), *p* < 0.001, Fig. 2A). Serum MDA levels were also significantly higher in the patients with PSCI compared with the non-PSCI group (3.6 (2.8–5.6) vs. 2.3 (1.7–3.0), *p* < 0.001, Fig. 2B). There were no significant differences in MDA (*p* = 0.245) or 8-OHdG (*p* = 0.145) levels between patients with mild cognitive impairment and patients with moderate to severe impairment.

There was a significant negative correlation between serum 8-OHdG levels and MMSE score, both in the illiterate patients (r = −0.401, *p* = 0.005) and in patients whose education level was limited to primary school (r = −0.282, *p* = 0.011). No correlation was found between MMSE scores and serum 8-OHdG levels in the total stroke patients (r = −0.140, *p* = 0.052) or in patients who attained an education level of secondary school or above (r = −0.192, *p* = 0.128). In the PSCI group (illiterate excluded), no correlation was found between MMSE scores and serum 8-OHdG levels (r = 0.007, *p* = 0.956).

Similarly, there was a significant negative correlation between serum MDA levels and MMSE scores in the total stroke patients (r = −0.252, *p* < 0.001), illiterate patients (r = −0.531, *p* < 0.001), and in patients with an educational level of secondary school or above were primary school (r = −0.542, *p* < 0.001). No correlation was found between MMSE scores and serum MDA levels in patients whose education level was primary school only (r = −0.216, *p* = 0.052). In the PSCI group (illiterate excluded), no correlation was found between MMSE scores and serum MDA levels (r = 0.056, *p* = 0.633).

**Figure 3:**
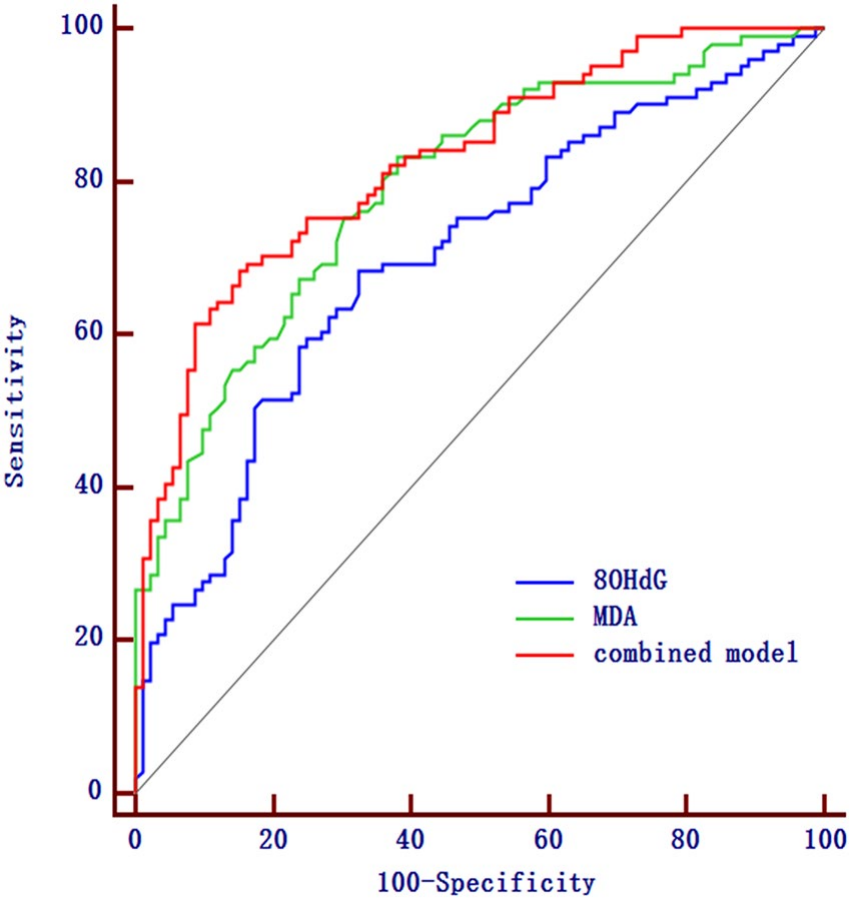
Diagnostic accuracies of 8-OHdG and MDA in discriminating PSCI patients from those without cognitive impairment in stroke patients. The combined model included 8-OHdG and MDA. Abbreviation: PSCI, post-stroke cognitive impairment; SE, sensitivity; SP, specificity; ROC, receiver-operating characteristic; AUC, area under curve; 95% confidence interval.

We found that both 8-OHdG and MDA had good diagnostic accuracy for PSCI (Fig. 3). ROC curve analysis was used to evaluate the usefulness of 8-OHdG to discriminate the presence of cognitive impairment and showed an area under curve (AUC) value of 0.700 (95% CI, 0.626–0.773; *p* < 0.001), and the optimal cut-off value for 8-OHdG as a diagnostic marker of stroke was 185.63 ng/L, which yielded a sensitivity of 68.3% and a specificity of 67.4%. The AUC value for MDA in discriminating the patients with cognitive impairment from the non-PSCI group was 0.793 (95% CI, 0.731–0.856; *p* < 0.001). The optimal cut-off for MDA was 2.59 nmol/ml, which showed a sensitivity of 83.2% and a specificity of 62.0%. Moreover, the combined model (8-OHdG and MDA) showed greater discriminatory ability (AUC, 0.826; 95% CI, 0.769–0.883; *p* < 0.001, Fig. 3) than either factor alone.

After adjusting for age, sex, BMI, hypertension, diabetes mellitus, CAD, hyperlipidaemia, smoking, drinking, BI score, NIHSS score, PSQI score, HAMD score, and drug use, higher 8-OHdG levels also independently predicted a cognitive impairment in the 4th week after IS (adjusted OR = 1.014, 95%CI (1.007–1.020), *p* < 0.001, Table 2). Similarly, higher MDA levels were also independently associated with PSCI after controlling for the same confounding variables mentioned above (adjusted odds ratio [95% confidence interval], 2.985 (1.990–4.478), *p* < 0.001, Table 2).

**Table 2:** Adjusted OR of cognitive impairment for serum levels of MDA and 8-OHdG in stroke patients.

Laboratory variables	Adjusted OR (95% CI)	Adjusted-*P* value
MDA (nmol/ml)	2.985 (1.990–4.478)	<0.001
8-OHdG (ng/L)	1.014 (1.007–1.020)	<0.001

The results are adjusted by age, sex, BMI, hypertension, diabetes mellitus, CAD, Hyperlipidemia, smoking, drinking, BI score, PSQI score, HAMD score, NIHSS score, drugs use (hypertension medicines prior to the ischemic events, statins and vitamin C at admission).

Abbreviations: 95% CI, 95% confidence interval; OR,odds ratio; BMI, body mass index; NIHSS, National Institutes of Health Stroke Scale; BI, Barthel Index.

Moreover, in multivariate analysis, there was an increased risk of PSCI associated with serum 8-OHdG levels above the cut-off (185.63 ng/L), after adjustment for the above variables (adjusted OR6.261, 95%CI2.766–14.176, *p* < 0.001). Serum MDA levels above the cut-off (2.59 nmol/ml) also increased the adjusted OR (OR [95% CI], 14.130 [5.250, 38.032], *p* < 0.001) after adjustment for the same variables.

## Discussion

The major findings of the present study were the following: (1) compared with the non-PSCI group, markedly increased serum levels of 8-OHdG and MDA were observed in patients with PSCI; (2) both 8-OHdG and MDA were negatively correlated with MMSE scores; (3) the levels of both 8-OHdG and MDA showed significant diagnostic accuracy in discriminating patients with PSCI from non-PSCI; and (4) elevated serum 8-OHdG and MDA were independently associated with PSCI at 1 month post-stroke.

The present study offers intriguing and possibly important findings on the role of 8-OHdG and MDA in PSCI. To the best of our knowledge, this is the first report of increased serum 8-OHdG (a marker of DNA oxidation) and MDA (a marker of lipid peroxidation) levels in patients with PSCI. Largely in accord with previous findings, our data support the role of oxidative stress in cognitive impairment. Elevated MDA levels were identified in patients with vascular or Alzheimer dementias compared to aged-matched control subjects^14^. Furthermore, MDA also increased in subjects with mild cognitive impairment^15, 16^, which suggests that lipid peroxidation is an early event in the progression of dementia. Similarly, individuals with AD or mild cognitive impairment had increased 8-OHdG levels in the brain, and in peripheral lymphocytes as well^17, 18^. Although there was an association between MDA or 8-OHdG and MMSE scores in the total stroke patients, unfortunately, we failed to find a significant correlation between the serum oxidative stress biomarker levels and MMSE scores in the PSCI group. Previous studies found that MMSE is negatively associated with MDA levels^19^. Compared with other cognitively impaired non-stroke patients, patients who suffer from cognitive impairment after stroke usually have additional complications, including vascular risk factors such as obesity, hypertension and diabetes, and smoking. The presence of these confounding factors in stroke may have an impact on our results to a certain extent. In addition, MMSE scores have a close relationship with the educational level of the subjects being tested. Therefore, future studies will be necessary to investigate whether MDA or 8-OHdG reflects the severity of cognitive impairment after stroke in a larger educated sample and in groups with more homogeneity, such as in smoking- and alcohol-free patients.

Cerebral ischaemia is a risk factor for vascular type dementias. The presence of hypoxia-ischaemia and vascular risk factors for vascular cognitive impairment are sufficient to trigger oxidative stress responses^20^. A large body of research indicates that oxidative stress is implicated in the pathogenesis of ischaemic brain injury through cell death pathways^21^. Lipid peroxidation induced by misfolding of a-synuclein may play an important role in the cellular mechanism of neuronal cell loss^22^. Products of lipid peroxidation may act as potential triggers of the p53 signalling pathway^23^ and cause disturbance to membrane organization and functional loss of mitochondrial and DNA^24^. Induction of oxidative DNA damage in the peri-infarct region in animal models of ischaemic stroke was demonstrated to be a contributory cause of secondary expansion of brain damage after permanent focal cerebral ischaemia and increased plasma levels of 8-OHdG were found to be associated with brain content of 8-OHdG^25^. The brain is exceedingly vulnerable to oxidative stress, due to a consumption of 20% of body oxygen, relatively high concentrations of iron content and readily peroxidizable lipids, as well as comparatively insufficient antioxidant defences^24^. Additionally, several clinical studies have shown elevated 8-OHdG and MDA are both markers of ischaemic brain injury and clinical outcome of ischaemic stroke^10, 26^. Moreover, Brea *et al*. reported that 8-OHdG was correlated with vascular recurrence in stroke patients who were not treated with statins^27^.

Increased 8-OHdG and MDA levels are both generally considered as indications of activated oxidative stress pathway. Oxidative stress may influence post-stroke cognitive impairment via different biological mechanisms. The disruption of the blood-brain barrier following ischaemia is thought of as a potentially important initiating factor in dementia^28^. Experimental animal studies suggest that possible involvement of oxidative stress is a causative factor in BBB dysfunction in stroke-pro spontaneously hypertensive rats^29^. Meanwhile, severe alteration of the BBB may be directly responsible for the extravasation of potent activators of free radicals and inflammatory products such as immunoglobulin G^30^ and fibrinogen^31^. Furthermore, in early human stroke matrix metalloproteinases that may participate in the damage to the white matter associated with vascular dementia^32^ were strongly linked to oxidative stress^33^. Additionally, oxidative stress may induce dysfunction of peroxisome proliferator-activated receptor (PPAR-γ), a situation that eventually leads to vascular ageing^34^, inflammation^35^, cerebrovascular white matter lesion and cognitive impairment^36^. Therefore, the results discussed above suggest that oxidative stress may play a critical role in the development of PSCI.

This study has some limitations. First, serum MDA and 8-OHdG levels were only evaluated for all patients within the first 24 h after admission. It may be essential to conduct a further longitudinal study measuring serum MDA and 8-OHdG levels at multiple time points after stroke and evaluating the predictive value of MDA and 8-OHdG at later times. Second, we excluded patients for whom we failed to measure serum levels of MDA and 8-OHdG, as well as those with both severe aphasia and with serious conditions, which might lead to an underestimation of the actual incidence of PSCI in the current study. Third, our study did not collect information on dietary status, which may influence the results. Fourth, considering our sample was measured at admission within 24 h, information concerning drug use during the days elapsed between the ischaemic event and hospitalization was not recorded.

In conclusion, we demonstrated that high serum MDA and 8-OHdG levels at admission are associated with the development of PSCI 1 month after acute ischaemic stroke and showed significant diagnostic accuracy in discriminating patients with PSCI from patients without cognitive impairment. Further studies are needed to verify this association. Our findings should be considered preliminary, and further clinical trials with MDA and 8-OHdG should be focused on identifying whether suppression of oxidative stress responses would be a novel and efficient tool against cognitive impairment in patients with ischaemic stroke.

## Methods

### Study subjects

The present study was performed in accordance with the ethical guidelines of the 1975 Declaration of Helsinki and was approved by the Medical Ethics Committee of the First Affiliated Hospital of Wenzhou Medical University, and all participants or their relatives provided written informed consent.

From October 2013 to September 2014, patients with their first-ever acute ischaemic stroke (IS), within 7 days of symptoms onset, were consecutively recruited from the Stroke Unit of the First Affiliated Hospital of Wenzhou Medical University.

The inclusion criteria were (1) age 18–80 years, (2) the ability and willingness to provide informed consent, (3) acute stroke occurring within 7 days before admission, and (4) a diagnosis of acute ischaemic stroke was verified from computed tomography (CT) or magnetic resonance imaging (MRI) at the time of admission. The following exclusion criteria were applied: (1) any history of stroke; (2) primary haemorrhagic stroke; (3) pre-stroke diagnosis of dementia or significant cognitive impairment; (4) significant neurological illness other than stroke, such as Parkinson’s disease; (5) severe hepatic, renal, or thyroid diseases or heart failure, respiratory failure or other organ failure; (6) chronic inflammatory, autoimmune or haematologic diseases; (7) history of depression (clinical diagnosis or previous treatment) or other psychiatric disorders; (8) severe aphasia or dysarthria or hearing impairment that might influence cognitive examination; (9) history of nootropics or antipsychotic drugs; and (10) patients who failed to measure serum 8-OHdG or MDA at admission.

### Assessments

Cognitive function evaluation was performed by the time of follow-up at 1 month by trained psychiatrists who were blind to the clinical presentations, examinations and laboratory results of the stroke patients, using the Chinese version of the Mini-Mental State Examination (MMSE). MMSE is a well-known questionnaire to rate the severity of cognitive impairment. A lower score indicates more severe cognitive impairment. PSCI was defined by a MMSE score ≤17 points (illiterate), ≤20 points(education level of primary school), or ≤24 points (education level of secondary school or above) respectively. Moreover, PSCI must be judged to be the consequence of stroke. Cognitive impairment severity was classified for those patients that were not illiterate by the following severity range for the MMSE: mild cognitive impairment (20–24), moderate cognitive impairment (10–19) and severe cognitive impairment (0–9).

Stroke severity was assessed using the National Institutes of Health Stroke Scale (NIHSS)^37^ on admission for all patients. Stroke aetiology was defined according to TOAST (Trial of Org 10172 in Acute Stroke Treatment)^38^ criteria by experienced clinicians at the time of patient admission. The infarct volume was evaluated in 120 patients using the initial DWI lesion volume and calculated as the sum of the infarction area of every slice multiplied by the slice thickness^39, 40^.

Additionally, we assessed the post-stroke functional impairment at discharge using the Barthel Index (BI; the scores range from 0 to 100, with lower scores indicating a more severe impairment)^41^. Depressive symptoms at discharge were evaluated based on the 17-item Hamilton Depression Scale (17-HAMD)^42^. Sleep quality of the patients was evaluated by Pittsburgh Sleep Quality Index (PSQI)^43^.

### Baseline clinical data collection

Information regarding demographic data (age, sex, body mass index [BMI], years of education), and vascular risk factors (hypertension, diabetes mellitus, hyperlipidaemia, coronary artery disease [CAD], smoking habit, and alcohol use) were collected from standardized questionnaires, which were conducted in face-to-face interviews by trained physicians.

### Blood collection and laboratory testing

Peripheral blood samples were obtained after admission within 24 h. A laboratory technician blinded to all clinical data processed all the samples. Serum samples for the determination of 8-OHdG and MDA were stored at −80 °C before being assayed.

Serum 8-OHdG levels were measured using a commercially available enzyme-linked immunosorbent assay (ELISA) kit. The inter-assay and intra-assay coefficients of variation (CVs) were 9% and 11%, respectively, and the mean minimum detectable dose (MDD) for human 8-OHdG was 10 ng/L.

Serum MDA levels were measured spectrophotometrically at 532 nm, with the assay detection limit was 0.5 nmol/ml. The inter- and intra-assay coefficient of variation (CV) was 4.11% and 3.5%, respectively.

### Statistical analysis

Continuous variables with a normal distribution are presented as the means ± standard deviations, and variables with a skewed distribution are expressed as medians (interquartile range). Categorical data are presented as frequency. For univariate analysis, differences in the clinical data were analysed by Student’s t-test, the Mann-Whitney U test, Kruskal-Wallis statistic and Pearson’s chi-square statistic, as appropriate. Correlations were assessed using the Spearman correlation test and Partial correlation. Receiver-operating characteristic (ROC) curve analysis was used to determine diagnostic accuracy, and the cut-off point was calculated according to the Youden index^44^. The influence of serum MDA or 8-OHdG on PSCI was estimated by logistic regression analysis, adjusted for potential influencing factors regarded as clinically relevant. Statistical analysis was performed using Prism 5 (GraphPad software, San Diego, CA) and SPSS 20.0 (SPSS Inc., Chicago, IL, USA). Receiver operative characteristics (ROC) curves were constructed through MedCalc 12.7 (MedCalc Software). Two-tailed *p*-values < 0.05 were considered statistically significant.

### Data availability statement

All data are fully available without restriction.

## Electronic supplementary material

Supplementary information
