# Supplementary material for: High Serum Levels of Malondialdehyde and 8-OHdG are both Associated with Early Cognitive Impairment in Patients with Acute Ischaemic Stroke
Source: Sci Rep. 2017 Aug 25;7:9493. doi: 10.1038/s41598-017-09988-3 (PMC5573400; doi:10.1038/s41598-017-09988-3)
Supplement: Supplementary file 1 — Supplementary information [file 41598_2017_9988_MOESM1_ESM.pdf]

Title: High Serum Levels of Malondialdehyde and 8-OHdG are both Associated with Early Cognitive Impairment in Patients with Acute Ischaemic Stroke

Authors: Zhihua Liu，Yuntao Liu, Xinjie Tu, Huiping Shen, Huihua Qiu, Huijun Chen, Jincai He*

Supplementary Fig. S1. Serum 8-OHdG and MDA levels according to the number of days elapsed from stroke symptoms onset.

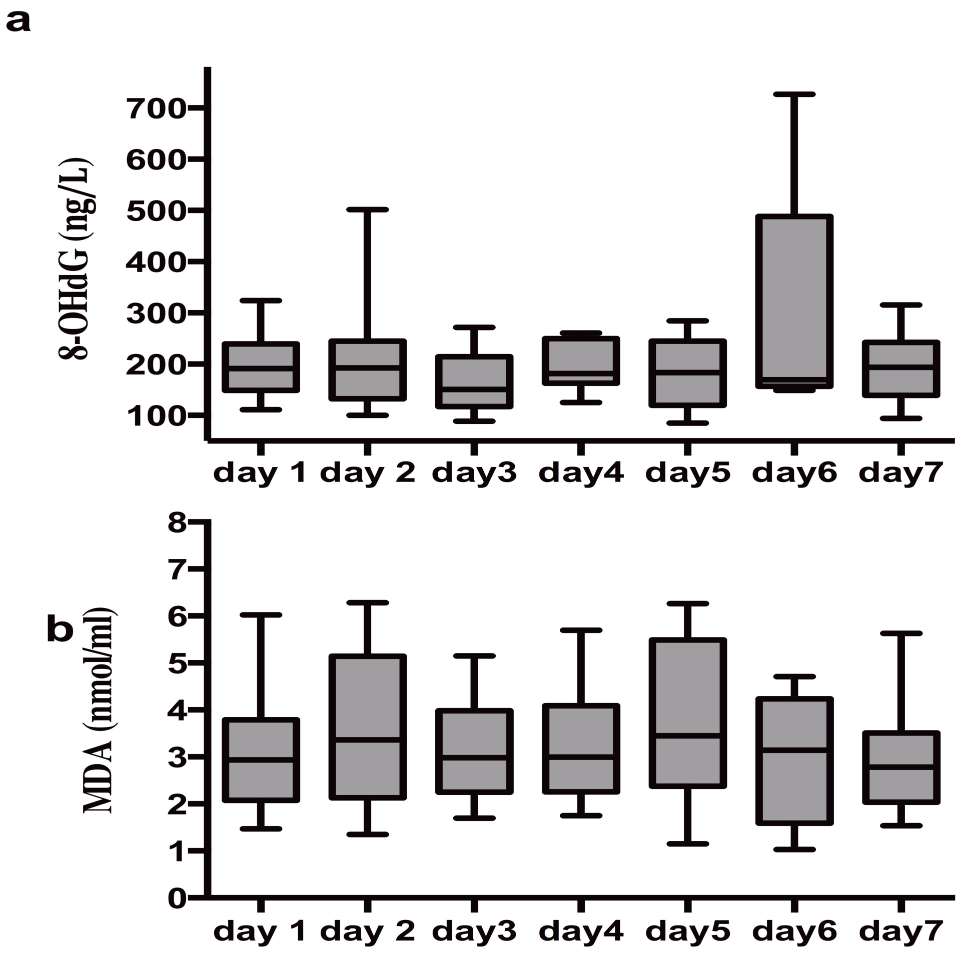

(A)8-OHdG levels, (B) MDA levels.

In the box-and-whisker plots, the horizontal line in the middle of each box indicates the median value; the lower and upper ends of the box represent the 25th and 75th percentiles, and the peripheral lines extending to the outer fences represent 10th and 90th percentiles, respectively. Abbreviation: 8-OHdG, 8-hydroxydeoxyquanosine; MDA, malondialdehyde.

Supplementary Table S1. Baseline characteristics of patients who dropped out of follow-up at 1-month interview

Baseline characteristics	Follow-up patients (n=193)	Loss of follow-up patients (n =47)	*P*-value
Gender, (M/F)	121/72	29/18	0.900
Age, yr, median (IQR)	63 (55-70)	60(52-66)	0.141
Years of education (years)	4 (0.5-7)	5 (1-8)	0.782
Education level,n(%)			0.996
Illiterate	48 (24.9)	11 (22.9)	
Primary school	81 (41.9)	21 (44.7)	
Secondary school or above	64(33.1)	15 (31.9)	
SBP (mmHg)	154.9±22.21	148.87±20.60	0.098
DBP (mmHg)	82.7±12.66	79.57±11.60	0.130
BMI, kg/m2	24.2 (22.0-26.0)	24.6 (21.8-27.1)	0.415
Stroke etiology,n(%)			0.775
Cardioembolism	10 (5.2)	3 (6.4)	
Atherosclerosis	174 (90.2)	41 (87.2)	
Small vessel occlusion	8 (4.1)	3 (6.4)	
Other undetermined etiology	1 (0.5)	0	
Vascular risk factors, n (%)			
Hypertension	124 (64.2)	32 (68.1)	0.621
Diabetes mellitus	64 (33.2)	14 (29.8)	0.658
Coronary artery disease	6 (3.1)	2 (4.3)	1.100
Hyperlipidemia	129 (66.8)	26 (55.3)	0.139
Smokers	81 (42.0)	19 (40.4)	0.847
Alcohol consumers	76 (39.4)	14 (29.8)	0.223
NIHSS score at admission	2 (1-4)	2 (1-4)	0.251
BI score at discharge	93 (60-100)	90 (55-100)	0.846

PSQI score at discharge	4 (3-7)	5 (2-6)	0.644
HAMD-17 score at discharge	5 (2-8)	5 (2-6)	0.761
MDA (nmol/ml)	3.0 (2.2-4.0)	3.2 (1.3-3.9)	0.785
8-OHdG (ng/L)	188.3 (137.6-238.8)	207.9 (144.9-249.9)	0.142

Continuous variables are expressed as the mean ± standard deviation (SD) or the median (interquartile range). Categorical values are given as frequencies (percentages).Abbreviation: BMI, body mass index; SBP, Systolic blood pressure; DBP, Diastolic blood pressure; NIHSS, National Institutes of Health Stroke Scale; BI, modified Barthel Index; PSQI, Pittsburgh Sleep Quality Index;HAMD-17, Hamilton depression rating scale 17-item; MDA, malondialdehyde ; 8-OHdG, 8-hydroxydeoxyquanosine; PSCI, Post-stroke cognitive impairment.

Supplementary Table S2. Serum MDA and 8-OHdG levels, according to the number of days elapsed from stroke symptoms onset.

	Day 1 (n=53)	Day2 (n=34)	Day3 (n=28)	Day4 (n=19)	Day5 (n=13)	Day6 (n=5)	Day7 (n=41)	*p*
MDA (nmol/ml)	2.9 (2.0-3.8)	3.4 (2.1-5.1)	3.0 (2.2-4.0)	3.0 (2.3-4.1)	3.5 (2.4-5.5)	3.1 (1.6-4.2)	2.8 (2.1-3.5)	0.763
8-OHdG (ng/L)	191.2 (148.8-239.7)	192.7 (132.6-245.0)	150.4 (117.2-214.5)	181.8 (162.7-249.9)	183.5 (119.8-245.0)	169.4 (56.9-488.5)	193.6 (139.3-242.8)	0.408

Continuous variables are expressed as the median (interquartile range).

The *P* values reflect comparisons between days elapsed from stroke symptoms onset to hospital admission.

Supplementary Table S3. Infarct volume and lesion location of patients for whom DWI data were available

	PSCI Patients (n =62)	Non-PSCI Patients (n =58)	*P* value
Infarct volume	1599.82(701.78-7725.20)	1175.49(413.67-2710.17)	0.039
Lesion location, n (%)			0.754
Left hemisphere	32 (31.7)	26 (28.3)	
Right hemisphere	25(24.8)	27 (29.3)	
Bilateral hemispheres	5(5)	5(5.4)	
Thalamus	7 (11.2)	10 (17.2)	0.350
Frontal lobe	12 (19.4)	8 (13.8)	0.414

The *P* values reflect comparisons between PSCI group and Non-PSCI group.

Supplementary Table S4. Comparisons of serum MDA and 8-OHdG levels in different infarct volumes

	V1	V2	V3	V4	*P*
MDA	2.9	2.8	2.6	3.0	0.697
(nmol/ml)	(2.1-3.8)	(2.2-4.0)	(1.7-4.1)	(2.6-3.7)	
8-OHdG	182.2	192.1	166.4	173.5	0.912
(ng/L)	(119.8-224.8)	(146.8-235.2)	(137.6-245.7)	(139.5-2377.3)	

Patients who have undergone DWI were divided into four groups according to infarct volume. V1 group: infarct volume <599.9 mm3; V2 group: 599.9 mm3 ≤infarct volume < 1411.3 mm3, V3 group: 1411.3 mm3 ≤infarct volume < 4194.3 mm3, V4 group: infarct volume ≥4194.3 mm3.

The *P* values reflect comparisons of serum MDA and 8-OHdG levels in different lesion volumes.

Supplementary Table S5. Serum MDA and 8-OHdG levels in various infarction locations of left, right or bilateral hemisphere

	Left hemisphere	Right hemisphere	Bilateral hemispheres	*P*
MDA	3.1(2.4-4.6) [a]	2.6 (1.8-3.4)	2.6 (2.0-3.3)	0.036[b]
8-OHdG	175.1 (121.8-226.8)	192.4 (150.7-240.1)	167.8 (99.8-188.3)	0.158

[a]$P < 0.05$:compared with Right hemisphere

[b]$P < 0.05$:comparsion of serum MDA and 8-OHdG levels in various infarction locations of left, right or bilateral hemisphere

Supplementary Table S6. Drugs employed of the studied subjects at admission.

Drugs	PSCI Patients (n ＝101)	Non-PSCI Patients (n=92)	P value
Statins use, n (%)	97 (96.0)	90 (97.8)	0.765
Alprostadil injection, Cinepazide Maleate injection or vinpocetine injection	101(100)	92 (100)	
Aspirin or clopidogrel use, n,(%)	101 (100)	92 (100)	
Hypertension medicine use, n (%)	52 (51.5)	42 (45.7)	0.418
Diabetes medicine use, n (%)	24 (23.8)	26 (28.3)	0.476
Butylphthalide use，n(%)	39 (38.6)	37 (40.2)	0.820
Vitamin C injection use, n (%)	8 (7.9)	4 (4.3)	0.305
Mannitol use, n (%)	5 (5.0)	3 (3.3)	0.821
Ginkgo Leaf Extract and Dipyridamole injection use, n (%)	9 (8.9)	5 (5.4)	0.352
Urinary Kallidinogenase use, n (%)	4(4.0)	3 (3.3)	1.000
Folic acid use, n(%)	6 (5.9)	5 (5.4)	0.880
